# Implementation barriers to integrating exercise as medicine in oncology: an ecological scoping review

**DOI:** 10.1007/s11764-021-01080-0

**Published:** 2021-09-12

**Authors:** Mary A. Kennedy, Sara Bayes, Robert U. Newton, Yvonne Zissiadis, Nigel A. Spry, Dennis R. Taaffe, Nicolas H. Hart, Daniel A. Galvão

**Affiliations:** 1 Exercise Medicine Research Institute, Edith Cowan University, 270 Joondalup Drive, Joondalup, Perth, WA 6027 Australia; 2 School of Medical and Health Sciences, Edith Cowan University, 270 Joondalup Drive, Joondalup, Perth, WA 6027 Australia; 3 School of Nursing, Midwifery and Paramedicine, Australian Catholic University, Fitzroy, VIC Australia; 4 School of Nursing and Midwifery, Edith Cowan University, Perth, WA Australia; 5 GenesisCare, Perth, WA Australia; 6 Cancer and Palliative Care Outcomes Centre, Queensland University of Technology, Brisbane, QLD Australia

**Keywords:** Cancer, Physical activity, Organizational change, Barriers, Chemotherapy, Radiotherapy

## Abstract

**Purpose:**

While calls have been made for exercise to become standard practice in oncology, barriers to implementation in real-world settings are not well described. This systematic scoping review aimed to comprehensively describe barriers impeding integration of exercise into routine oncology care within healthcare systems.

**Methods:**

A systematic literature search was conducted across six electronic databases (since 2010) to identify barriers to implementing exercise into real-world settings. An ecological framework was used to classify barriers according to their respective level within the healthcare system.

**Results:**

A total of 1,376 results were retrieved; 50 articles describing implementation barriers in real-world exercise oncology settings were reviewed. Two hundred and forty-three barriers were identified across all levels of the healthcare system. Nearly 40% of barriers existed at the organizational level (n = 93). Lack of structures to support exercise integration and absence of staff/resources to facilitate its delivery were the most common issues reported. Despite the frequency of barriers at the organizational level, organizational stakeholders were largely absent from the research.

**Conclusions:**

Implementing exercise into routine cancer care is hindered by a web of interrelated barriers across all levels of the healthcare system. Organizational barriers are central to most issues. Future work should take an interdisciplinary approach to explore best practices for overcoming implementation barriers, with organizations as a central focus.

**Implications for Cancer Survivors:**

This blueprint of implementation barriers highlights critical issues that need to be overcome to ensure people with cancer have access to the therapeutic benefits of exercise during treatment and beyond.

**Supplementary Information:**

The online version contains supplementary material available at 10.1007/s11764-021-01080-0.

## Introduction

An important paradigm shift regarding the role of exercise in oncology care has taken place. Prior to the 1980s, when the potential for exercise to alleviate negative side effects of cancer treatment was first investigated [1], standard medical advice was to prescribe “rest therapy” because any unnecessary activity was considered potentially harmful during treatment [2, 3]. In the decades since, hundreds of studies have demonstrated the safety and efficacy of exercise for people living with and beyond cancer [4]. Researchers suggest that exercise has an important therapeutic role in preparing patients for surgery and treatment [5, 6], managing treatment-related side effects [7] and improving treatment tolerability [8], with emerging evidence of a potential role for exercise to enhance the effectiveness of treatment [9–11]. Based on this robust evidence base, national and international organizations have developed prescriptive guidelines to assist exercise professionals in harnessing the therapeutic benefits of exercise according to a patient’s cancer type and treatment regimen [12, 13]. Further, the potential role for exercise during treatment has become so compelling that the American College of Sports Medicine (ACSM), a world leading authority on exercise, issued a call for clinicians and other key stakeholders to take action in creating an infrastructure within healthcare to facilitate the incorporation of exercise into routine care for people with cancer [14]. While this evidence base is crucial, it is not meaningful if patients are not offered or cannot access exercise during treatment.

While best practice guidance continues to be refined for healthcare providers regarding exercise screening, referrals and programming [12–17], exercise is not routinely incorporated as a component of cancer care. Dissemination efforts appear to have successfully informed oncology clinicians and patients that exercise can be a component of care [12, 13]; however, few meaningful changes in behaviour have occurred as a result of this increased awareness. Researchers have reported that oncologist engagement in exercise counselling is low (13 to 27%) [18, 19] and largely unchanged from 15 years ago [20]. Patient engagement in physical activity during treatment also remains suboptimal (< 50%) across multiple cancer types [13, 21–23]. Given the robust evidence base that has been generated for the therapeutic benefits of exercise during treatment, it is critical to start investigating how to best translate exercise oncology research into practice to ensure that patients are receiving optimal care.

The first step to guide future translation efforts is to identify what is stopping the successful delivery of exercise in clinical care (i.e. implementation barriers). We have defined implementation barriers as those that exist outside of an individual’s personal preferences and represent issues that impede the ability to offer or access exercise, rather than issues that influence engagement and participation. For example, implementation barriers for potential referrers are things that could complete the statement “I wanted to offer exercise to my patient but could/did not because…” and for patients, “I wanted to engage in exercise during treatment but could/did not because…”.

Barriers to the provision of exercise medicine in cancer treatment settings have been well described in the literature [20, 24, 25]; however, these barriers have not been evaluated from an implementation perspective. The aim of this review is to help address the translation gap by summarising what is known about implementation barriers in exercise oncology settings. The review was framed using an ecological perspective [26], which allowed for the identification of barriers across the multiple levels of healthcare. The resulting map of the literature will be of interest to those looking to engage in exercise oncology implementation research and practice and will provide direction for future research in this emerging field.

## Methods

A systematic scoping review was chosen to synthesize the literature related to the barriers of implementing exercise into oncology care. The exploratory nature of this methodology was deemed most appropriate to achieve the goal of providing a comprehensive perspective of the implementation challenges for exercise oncology. Our approach was informed by the PRISMA-ScR (Preferred Reporting Items for Systematic reviews and Meta-Analyses extension for Scoping Reviews) checklist [27]. Grol and Wensing’s ecological framework was used to guide the review [26]. The framework considers potential barriers across six distinct levels of a healthcare system to help describe the interaction between individuals and the environment within the system (Table 1) [26]. Importantly, the framework notes implementation failures often involve factors across multiple levels.

**Table 1:** Barriers to change at different levels of healthcare from Grol and Wensing 2004 [26]

Level	Barriers/incentives
Innovation	Advantages in practice, feasibility, credibility, accessibility, attractiveness
Individual professional	Awareness, knowledge, attitude, motivation to change, behavioural routines
Patient	Knowledge, skills, attitude, compliance
Social context	Opinion of colleagues, culture of the network, collaboration, leadership
Organizational context	Organization of care processes, staff, capacities, resources, structures
Economic and political context	Financial arrangements, regulations, policies

Inclusion criteria were determined utilising the PCC (Population, Context, Concept) framework [28] (Table 2). We limited our search to programs connected with clinical settings, as the aim of this review is to inform efforts to incorporate exercise into routine healthcare. Additionally, our search was limited to studies published since 2010 because this was when the first set of international exercise guidelines for oncology were created [29], which raised awareness of potential for exercise to be integrated into oncology practice. Only papers reporting original research studies were included.

**Table 2:** Inclusion and exclusion criteria for scoping review of the literature

Category	Inclusion criteria	Exclusion criteria
Population	People with an experience related to implementation of exercise into cancer care, including but not restricted to: People receiving the program People living with and beyond cancer People referring into an exercise program: Oncologists Nurses General practitioners People delivering an exercise program: Exercise physiologists Physiotherapists People implementing an exercise program: Hospital administrators Program administrators People designing exercise programs: Researchers	Age ≤ 18 years
Concept	Literature describing barriers to accessing or implementing exercise programs	Barriers unrelated to issues of implementation (e.g. personal motivation) Barriers that were anticipated, not experienced Reported exercise preferences, not experienced barriers
Context	Outpatient clinical care	Exercise programs that are not linked into healthcare, either via clinician referral or location (e.g. co-located) In-patient exercise programs Non-real-world programs (e.g. clinical trials)
Study design (and study feature)	Any design inclusive of original research English language only Published 2010–2020	Reviews or meta-analyses Guidelines or position stands Program descriptions Insufficient detail to determine any relevant study content

### Search strategy

The PCC inclusion criteria informed the search strategy (Supplemental Table 1). Search terms were created based on the concept (implementation barriers of exercise), context (real-world outpatient clinical care) and population of interest (people with an experience related to implementation of exercise into cancer care) in consultation with professional medical library staff. We specified that terms related to exercise and cancer should be included in article titles as our preliminary search indicated that this would help to limit results to the most relevant results. Search term sets were combined (using AND) and exploded as appropriate using the truncation (*). Language (English) and time frame limitations (publication date after January 1, 2010) were also set.

**Fig. 1:**
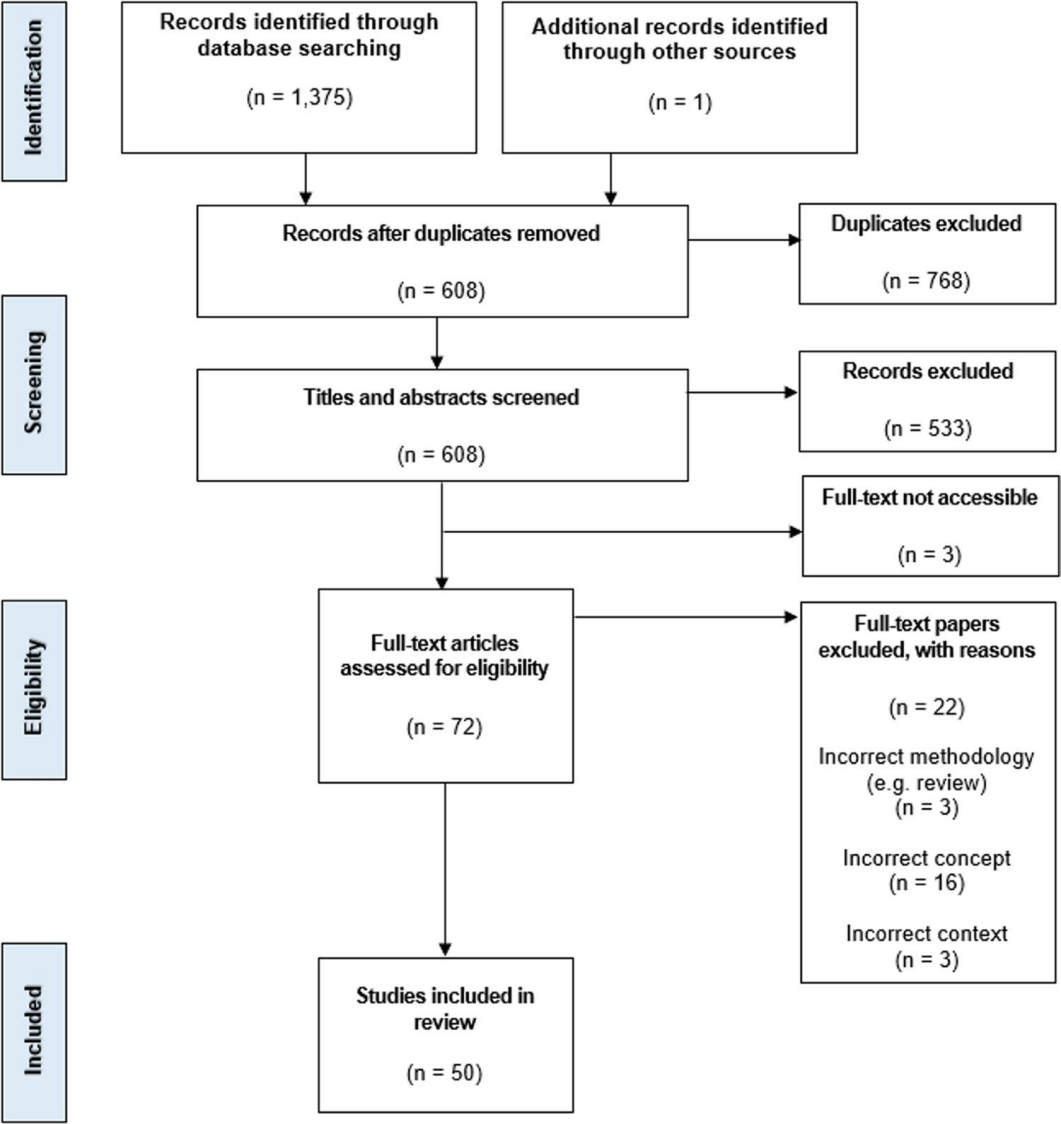
PRISMA-ScR flow diagram for systematic scoping review of implementation barriers in exercise oncology

Between May and July 2020, two authors (MAK, SB) systematically searched the MEDLINE (n = 335), CINAHL (n = 173), PsychINFO (n = 75), PubMed (n = 520) and Web of Science (n = 272) databases for relevant studies using our search terms. We imported the results of all searches into EndNote and maintained relevant bibliographic databases using the process recommended by Peters (2017) [30]. The Scopus database was also searched and yielded a small number of results (n = 13). These results were checked against the bibliographic database created from the previous searches; all were duplicates so were not imported. One additional paper was identified through reference lists of relevant articles and was added to the database. This yielded a total of 1376 articles (Fig. 1). Duplicates were removed, and titles and abstracts of these results were screened (MAK) to exclude any that did not obviously meet our PCC criteria (e.g. pediatric patient population, review article). Results that presented ambiguous information or did not describe key details (e.g. description of the population) remained included to ensure relevant results were not missed. Full text for three articles could not be accessed after multiple attempts, so were removed from the results. Full text for the 72 articles deemed potentially relevant were read (MAK, SB) to determine those that met criteria for PCC inclusion. Fifty met the inclusion criteria and were included in the review.

### Data extraction and synthesis

Data extraction was conducted for all articles meeting selection criteria using a matrix developed by authors (MAK, SB). Matrix development was guided by the framework proposed by Miles, Huberman and Saldana [31] to identify and display information in a way that is helpful to answer the research questions being asked. The matrix structure clearly defined integral methodological components relevant to each study’s PCC.

Two authors (MAK, SB) conducted the analysis of the matrix. In the first stage, each article was carefully reviewed, and all relevant data were extracted and entered into the review matrix. Allocation of data into categories was discussed, and full consensus was reached through this process. In the second stage, data relevant to implementation barriers were identified and grouped into themes. In the final stage, the themes were classified into one of six implementation influences: innovation, individual professional, patient, social context, organizational context or economic and political context. In some instances, a barrier aligned with more than one influence. To avoid repetition, each barrier was allocated to the influence level that represented the largest impact within the ecological framework. Verbatim quotes from participants of the included studies are provided throughout to help illustrate each theme.

## Results

### Study and participant characteristics

The studies were primarily exploratory in nature (Table 3). Year of publication ranged from 2010 to 2020, with the majority (n = 35, 70%) published between 2017 and 2020. Most studies (n = 33, 66%) used a qualitative design. Twelve (24%) used a quantitative design and five (10%) incorporated mixed methods. The studies were conducted in 12 countries of which three were dominant (Canada n = 11, 22%; Australia n = 9, 18%; USA, n = 9, 18%) accounting for 58% of the total.

**Table 3:** Study characteristics

Author and study year	Country	Study design	Stakeholder group (n)	Cancer type	Context	Concept
Agasi-Idenburg et al. 2020 [70]	Netherlands	Qualitative	Patients (n = 15) Healthcare providers (n = 9)	Colorectal	Netherlands Cancer Institute	Barriers for preoperative exercise programs for older patients scheduled for colorectal cancer surgery
Beidas et al. 2014 [35]	USA	Qualitative	Healthcare providers (n = 19)	Breast	NCI designated comprehensive cancer centre and associated physical therapy clinic	Barriers to implementation of efficacious exercise intervention for breast cancer survivors experienced by referring oncology clinicians and physiotherapists who delivered the program
Blaney et al. 2010 [71]	Northern Ireland	Qualitative	Patients (n = 26)	Multiple	Regional Cancer Centre in Belfast	Barriers to exercise among patients with cancer related fatigue
Bourke et al. 2018 [55]	UK	Qualitative	Patients (n = 26) Healthcare providers (n = 38)	Prostate	National Health Service	Exercise referral and tailored exercise training embedded within prostate cancer care
Brunet et al. 2013 [74]	Canada	Qualitative	Patients (n = 9)	Breast	Healthcare system in Canada	Active breast cancer survivors’ perceptions of factors that influence their PA participation
Bultijnck et al. 2018 [60]	Belgium	Quantitative	Healthcare providers (n = 98)	Prostate	Hospitals across Belgium	Availability of hospital-based rehabilitation resources for prostate cancer patients
Cantwell et al. 2018 [36]	Ireland	Multi-method (Delphi method)	Healthcare providers (n = 91)	Multiple	Healthcare system across Ireland	Barriers to PA promotion for cancer survivors
Cantwell et al. 2020 [67]	Ireland	Qualitative	Patients (n = 41)	Multiple	Cancer support centre and community-based exercise rehabilitation program	Experiences of PA behaviour across the cancer journey
Cheville et al. 2012 [75]	USA	Qualitative	Patients (n = 20)	Lung	A single cancer treatment service	Perceived barriers for exercise and exercise-related instructions received from their professional caregivers
Culos-Reed et al. 2019 [65]	Canada	Qualitative	Patients (n = 11)	Prostate	Community-based lifestyle management program	Patient perspectives of the community-based TrueNTH Lifestyle Management program
Dalzell et al. 2017 [33]	Canada	Quantitative	Patients (n = 1,635)	Multiple	Segal Cancer Centre within the Jewish General Hospital, Montreal Quebec	Evaluation of implementation of rehabilitation and exercise oncology program (ActivOnco) within hospital setting
Dennett et al. 2017 [63]	Australia	Qualitative	Healthcare providers (n = 15)	Multiple	Australian oncology rehabilitation programs	Barriers to exercise program implementation
Dennett et al. 2020 [37]	Australia	Qualitative	Patients (n = 9) Healthcare providers (n = 25)	Multiple	Large, public, metropolitan health service	Barriers to implementing an exercise-based rehabilitation program in an acute setting for cancer survivors receiving treatment
Fernandez et al. 2015 [76]	Canada	Mixed	Patients (n = 30)	Multiple	Outpatient physiotherapy programs or established cancer support organizations in communities within Ontario	Barriers to exercise in individuals with cancer
Fitzpatrick et al. 2014 [62]	USA	Mixed	Healthcare providers (n = 50) Researchers (n = 84)	Multiple	Research and clinical settings across USA	What needs to happen for exercise to become part of standard care for cancer survivors once treatment ends
Fong et al. 2018 [24]	Canada	Qualitative	Healthcare providers (n = 27)	Breast	Regional cancer centres across Ontario	Factors affecting PA counselling in clinicians
Fong et al. 2018 [32]	Canada	N/A	N/A	Breast	Regional cancer centres across Ontario	Built environment scan of PA infrastructure
Granger et al. 2016 [38]	Australia	Qualitative	Healthcare providers (n = 17)	Lung	University of Melbourne-affiliated hospital networks	Barriers that influence clinicians’ translation of the PA guidelines into practice
Granger et al. 2019 [64]	Australia	Qualitative	Patients (n = 7)	Lung	Community around The University of Melbourne, Australia	Explore patient experiences of PA after a lung cancer diagnosis
Hardcastle et al. 2018 [39]	Australia	Qualitative	Patients (n = 20)	Multiple	State sponsored outpatient group exercise program in Western Australia	Factors influencing non-participation in structured exercise program for cancer survivors
Haussmann et al. 2018 [40]	Germany	Quantitative	Healthcare providers (n = 675)	Multiple	Outpatient oncology care settings across Germany	Structural barriers perceived as impeding by healthcare providers for promoting PA to patients
Haussmann et al. 2018 [56]	Germany	Qualitative	Healthcare providers (n = 30)	Breast, prostate, and/or colon	Outpatient and inpatient settings in Baden-Wuerttemberg, Germany	Influencing factors for healthcare providers’ PA promotion behaviour and reasons and mechanisms behind them
Höh et al. 2017 [41]	Germany	Quantitative	Patients (n = 905)	Multiple	Federal association of cancer self-help	Experience with PA in cancer
Hubbard et al. 2018 [73]	UK	Mixed	Patients (n = 32)	Breast	UK hospital serving rural and urban population	Acceptability and feasibility of post-surgery referral to existing community-based PA programs
IJsbrandy et al. 2019 [42]	Netherlands	Qualitative	Patients (n = 34)	Multiple	Hospitals in the Netherlands	Factors that influence the implementation of PA programs
IJsbrandy et al. 2020 [43]	Netherlands	Qualitative	Healthcare providers (n = 70)	Multiple	Dutch healthcare system	Factors affecting delivery of PA programming in a shared-care model
Kang et al. 2014 [72]	Republic of Korea	Quantitative	Patients (n = 427)	Colorectal	Shinchon Severance Hospital	Barriers to exercise in colorectal cancer patients and survivors
Karvinen et al. 2012 [44]	USA	Quantitative	Healthcare providers (n = 274)	Multiple	Oncology care settings across the USA	Barriers to PA promotion among oncology nurses
Kennedy et al. 2020 [45]	Australia	Qualitative	Patients (n = 119) Healthcare providers (n = 15)	Multiple	Private oncology outpatient clinic	Barriers to implementation of co-located exercise clinic
Keogh et al. 2014 [77]	Australia	Qualitative	Patients (n = 14)	Prostate	Private and public healthcare system	Barriers to PA in men with prostate cancer
Keogh et al. 2017 [46]	Australia and New Zealand	Quantitative	Healthcare providers (n = 119)	Multiple	Private and public oncology care across Australia and New Zealand	PA promotion barriers of oncology nurses
Ligibel et al. 2019 [47]	USA*	Quantitative	Healthcare providers (n = 812)	Multiple	International oncology practice	Practice patterns around assessment of body weight, PA, and nutrition and referrals to relevant programs to support behaviour change after a cancer diagnosis
Maxwell-Smith et al. 2017 [78]	Australia	Qualitative	Patients (n = 24)	Colorectal	St. John of God Hospital, Perth Australia	Explore colorectal survivors’ experiences and barriers towards PA among those with comorbidities
Mulcahy et al. 2018 [58]	Ireland	Qualitative	Organizational stakeholders (n = 24)	Multiple	Specialised cancer centres, public and private hospitals and palliative care settings across Ireland	Barriers to the provision of physiotherapy exercise rehabilitation services available to patients with cancer
Nadler et al. 2017 [18]	Canada	Quantitative	Healthcare providers (n = 120)	Multiple	Juravinski Cancer Centre in Hamilton, Ontario	Determine oncology care providers barriers to exercise discussion
O’Hanlon et al. 2014 [48]	Ireland	Quantitative	Healthcare providers (n = 84)	Multiple	Oncology care across Ireland	Identify barriers to prescribing exercise for cancer care
Park et al. 2015 [49]	Republic of Korea	Quantitative	Healthcare providers (n = 165)	Multiple	Oncology care across the Republic of Korea	Barriers to recommending exercise to cancer survivors
Patel et al. 2018 [69]	New Zealand	Qualitative	Healthcare providers (n = 16)	Prostate	Private and public practices in New Zealand	Influences on practitioners to not promote PA to their patients with prostate cancer
Perry et al. 2020 [61]	USA	Mixed	Patients (n = 61) Healthcare providers (n = 11)	Breast	University cancer centre in Pacific Northwest	Attitudes and beliefs regarding exercise counselling and structured exercise programs within cancer care
Roberts et al. 2019 [57]	UK	Qualitative	Healthcare providers (n = 19)	Breast, prostate, colorectal	Breast, prostate and colorectal care across the UK	Perspectives on PA promotion
Rogers et al. 2019 [66]	USA	Qualitative	Healthcare providers (n = 14) Organizational stakeholders (n = 12) Community stakeholders (n = 4)	Mixed	Rural community setting	Identify constructs relevant to implementation of evidence-based PA behaviour change interventions for rural women cancer survivors from an organizational perspective
Romero-Elias et al. 2020 [50]	Spain	Qualitative	Patients (n = 10) Healthcare providers (n = 10)	Colorectal	Oncology unit of a Spanish hospital	Barriers patients perceive to participate in PA during chemo + views of physicians
Santa Mina et al. 2015 [51]	Canada	Qualitative	Organizational level (n = 13)	Multiple	Cancer exercise programs offered across Canada	Understand process of program implementation and barriers to program success
Shea et al. 2020 [52]	Canada	Qualitative	Healthcare providers (n = 20) Organizational stakeholders (n = 10)	Multiple	Atlantic Canadian cancer centres and organizations with PA programs tailored for cancer survivors	Explore barriers to program implementation
Smaradottir et al. 2017 [53]	USA	Qualitative	Patients (n = 20) Healthcare providers (n = 9)	Multiple (excluding breast)	Gundersen cancer centre	Barriers to implementing an exercise program during cancer treatment
Smith et al. 2017 [79]	UK	Qualitative	Patients (n = 19)	Multiple	UK cancer centres	Explore cancer survivors’ potential barriers to exercise
Smith-Turchyn et al. 2016 [54]	Canada	Qualitative	Healthcare providers (n = 24)	Breast	Outpatient cancer centres across southwestern Ontario	Barriers of exercise promotion for women with breast cancer
Spost 2015 [59]	USA	Qualitative	Healthcare providers (n = 36)	Breast		Factors that prevent from recommending exercise
Sutton et al. 2017 [68]	UK	Qualitative	Patients (n = 16) Healthcare providers (n = 10)	Prostate	Tertiary referral hospital urology department southwest of the UK	Experiences of provision of PA advice following diagnosis of and treatment for prostate cancer
Tomasone et al. 2017 [34]	Canada	Qualitative	Healthcare providers Organizational stakeholders Community stakeholders (n = 124)**	Multiple	Cancer programs in Ontario	Determine strategies for implementation of ‘exercise for people with cancer’ guideline

^*^Study was conducted at an international conference held in the USA, though participants practiced around the world

^**^Participant breakdown not provided

*NCI* National Cancer Institute, *PA* physical activity

Study participants represented five stakeholder groups: patients, healthcare providers (HCPs), organizational representatives, community representatives and researchers. Most studies (n = 36, 72%) included participants from a single stakeholder group. Eleven (22%) incorporated a combination of two or more groups, and two studies did not include any participants due to the nature of their design (i.e. built environment scan and service audit) (n = 2, 4%) [32, 33]. A total of 1895 patients were represented across nearly half (n = 23, 46%) of the studies (Supplemental Table 2). Studies included participants with a variety of tumour types. Breast and colorectal cancer accounted for 30% of the total (breast n = 579, 16%; colorectal n = 479, 14%). The phase of survivorship (i.e. pre- and/or active treatment vs. post-treatment) of patients was reported in 15 studies representing 709 patients. Within those, 34% of patients were receiving treatment during their study participation. Two thousand eight hundred and forty-six HCPs participated across 31 (62%) studies. Physicians (51%, n = 1455) and nurses (38%, n = 1085) were the most represented, with allied health professionals (e.g. physiotherapists, radiation therapists) accounting for 11% of HCPs (n = 306). Physiotherapists represented over half of the allied health professionals (61%, n = 186). Stakeholders from organizations, research and communities were represented in five studies. One study [34] included all three stakeholder groups but did not breakdown the composition of the 124 participants. The remaining four studies included 59 organizational stakeholders (e.g. hospital administrators, program coordinators), 56 researchers, and four community partners.

### Barriers to program implementation

**Fig. 2:**
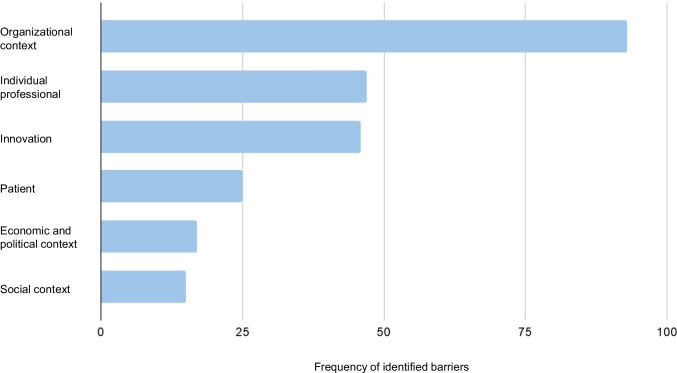
Frequency of barriers identified at each level of healthcare

A total of 243 implementation barriers were extracted from 50 studies. Barriers were found across all levels of the framework (Fig. 2; Supplemental Table 3). They are described below in order of frequency by level of healthcare.

### Organizational context

Ninety-three barriers were described in 38 studies [18, 24, 32–67]. Organizational context barriers focused on capacity, staff and resources and structures/organization of care processes.

#### Capacity

Capacity issues were expressed in 22 barriers across 19 studies [18, 24, 36, 38, 40, 42–44, 46–50, 53, 54, 57–59, 61]. The inability of HCPs to counsel, prescribe and refer patients to exercise in the time allotted for patient visits was highlighted. In a survey of oncology providers, 66 percent (n = 540) of respondents either strongly agreed or agreed that lack of time for counselling or to set up a referral was a barrier to providing lifestyle interventions to patients, including exercise [47]. One nurse made the point by saying simply, “the issue is just more time” [61] (p. 61). Workload pressures [61], concerns about the extra work [exercise counselling] would entail [43], and concern for the extra time necessary to complete in-clinic counselling would disrupt clinic flow [24] were raised as related issues.

#### Staff and resources

Thirty-four barriers described challenges with staffing and resources related to exercise. Limited resources to build exercise into care was described as a barrier in 20 studies [24, 33, 34, 36–38, 40, 42, 43, 47, 48, 51, 52, 54, 55, 58, 61–63, 66]. Resources included staff, funding and referral networks. A lack of staff with expertise in exercise programming was noted in five studies [37, 38, 40, 52, 62]. Funding to support qualified staff or purchase exercise equipment was noted in eight studies [24, 33, 34, 51, 52, 54, 62, 63], and a lack of exercise resources to refer to was described in four studies [36–38, 47]. A nurse summed up the consequence of inadequate exercise resourcing by saying: “It’s not worth bringing it up. You don’t plant the seed unless you can water it” (nurse) [37].

#### Structures and organization of care processes

Absence of an established pathway or structure to support the inclusion of exercise into care was raised 37 times in 24 studies [32, 34, 35, 37–39, 41, 43, 45, 46, 48, 50, 52–54, 56–58, 60, 62, 64–67]. Inadequate infrastructure to support a standard referral to exercise was described, including a lack of standard or effective referral pathway [35, 38, 45, 62] and the lack of an exercise expert as part of the core care team [34, 38, 39, 52, 62]. One study, which explored a state run non-profit exercise program designed for people with cancer, found that none of the participants were referred to the program by their treating oncology team [39]. Other structural issues noted were the challenge of managing referrals between separate locations [35, 45] and the absence of a system to collect physical activity information about patients [53].

Standard model of care processes were deemed “fragmented” [38] with HCPs describing processes as “reactive” rather than “preventative” [43], leaving no room for development of an exercise service. Physicians felt that it was impractical for them to advise on exercise, as they typically do not engage in long-term follow-up for patients [38]. Patients recognised this issue as well, describing follow-up care regarding exercise as inadequate [64, 67] representing a “gap in the cancer care pathway” [67].

### Individual professional

Forty-seven barriers were described in 23 studies [18, 34, 37, 38, 43, 44, 46–57, 62, 63, 68, 69]. Individual professional barriers were described regarding knowledge and attitude toward exercise.

#### Knowledge

Twenty-three barriers were highlighted in 16 studies [18, 34, 37, 38, 43, 48, 50, 52–57, 62, 63, 68]. A lack of knowledge was the most common barrier reported at the individual professional level with HCPs reporting insufficient knowledge to advise patients about exercise [18, 34, 37, 38, 43, 48, 50, 52–57, 62, 63, 68] or refer them to an appropriate resource [18, 37, 38, 43, 52]. A survey of 120 oncology care providers revealed that at least 77% (n = 85) rated their knowledge as “poor” regarding how to counsel based on exercise guidelines and knowing when, how and which patients to refer to a supervised exercise program [18], with only 13% (n = 16) providing specific information to patients. An oncologist described how their lack of knowledge resulted in vague advice for patients: “When patients ask me what they can do I say well just do whatever you want…” [37]. Specifically, a lack of understanding of appropriate guidelines [18, 57] or how to safely prescribe during treatment [48] were highlighted, as well as a lack of skill around behaviour change techniques [47, 55–57]. Patients reinforced this barrier, noting their doctors’ inability to provide meaningful exercise information (described in patient level barrier).

#### Attitude

Twenty-four barriers related to the attitude of HCPs incorporating exercise into care for people with cancer were described. There were three distinct concerns that emerged in this category. First, HCPs reported a perception of patients being uninterested or resistant to receiving exercise information in nine barriers across eight studies [24, 38, 44, 47, 48, 51, 52, 56] with some HCPs noting that patient characteristics influenced a willingness to offer exercise. For example, HCPs described hesitation referring patients to exercise who were previously inactive, elderly or undergoing treatment [24, 51, 56]. One general practitioner (GP) described this by saying: “I mean for some people, the idea to put 80-year old people on treadmills is close to torture…” [38].

Second, HCPs reported uncertainty about the safety and quality of exercise as a barrier eight times across eight studies [18, 24, 43, 46, 49, 51, 56, 57]. For example, within a sample of 167 oncologists, only ~ 40% agreed “exercise is safe” for patients [49]. A patient’s overall health and their ability to exercise during treatment were common concerns, with worries that exercise would cause “overexertion” or make a patient “even more weak” [56]. These safety concerns were reinforced by cancer exercise program coordinators who noted “a reluctance [of physicians] to refer patients because of safety concerns” as a barrier to their program’s success [51] (p. 380). Physicians also expressed a reluctance to refer to exercise programs because they could not be assured of their quality [24, 43].

Third, exercise was not deemed a priority during time constrained office visits for HCPs in seven barriers across five studies [52–54, 56, 69]. It was described as an “auxiliary” issue [69] (p. 35) that did not take precedence over other components of care [52, 53, 69], was overlooked because it is not a “thing of priority” for physicians [56] or was seen as someone else’s responsibility [69]. A medical oncologist made this point clearly, stating “…I feel that there are other people who can actually address [exercise], because the patient comes to see me for the expert opinion for the management of their cancer. The other auxiliary issues can be dealt with by other health professionals. No one else is going to give them the advice I can give as a medical oncologist” [69] (p. 35).

### Innovation

Forty-six barriers were described in 25 studies [24, 34, 36–39, 42, 43, 45, 51–57, 61–63, 65, 70–74]. Innovation level barriers were described across two major categories: advantages in practice and accessibility.

#### Advantages in practice

There was an indication that confidence about the advantage of exercise in clinical care is low for some HCPs; 10 barriers suggesting that clinicians were not aware of or did not believe in the benefits of exercise for patients were described in eight studies [34, 38, 43, 51, 52, 55, 56, 62]. Specifically, physicians deemed exercise not beneficial for specific groups of patients, such as those who are “already fit” [38], “elderly” [55] or undergoing chemotherapy [56]. These concerns were underpinned by HCPs’ view that the evidence to demonstrate the benefits of exercise for people with cancer was inadequate [38, 43, 62].

#### Accessibility

Thirty-six accessibility barriers related to cost, location and availability were identified across 21 studies. Eighty-one percent of HCPs (n = 48) in one study indicated that they either strongly agreed or agreed that patients “experienced or could experience poor access to programs (e.g. in terms of transport, cost, location, waiting lists)” [36].

The direct cost of an exercise program was highlighted as a barrier to participation by patients and to referral by HCPs, as described in 11 barriers across 11 studies [36, 38, 39, 43, 53, 54, 62, 71–74]. For patients, direct participation costs were a concern for unsubsidised programs such as fitness centres [39, 71, 73]. One recently diagnosed patient stated simply “I couldn’t afford to join a gym…” [71] (p. 1142).

Indirect patient cost issues, such as those associated with transportation, were also raised as concerns [39, 53, 57] and are related to the accessibility barrier of program location described in 14 barriers across 12 studies [36, 37, 39, 42, 45, 51, 53, 54, 57, 61, 63, 73]. Patients, HCPs and organizational stakeholders highlighted the location of a program as a deterrent to participation. Specific concerns included locations that required long travel times [39] or involved convenience issues such as a lack of parking [37, 45]. A breast cancer nurse specialist explained the challenge by saying: “it’s alright bringing up this about exercising, but how they’re going to get there, what’s the cost of it, err, I live on my own, you know, all these sorts of barriers that are put up” [57] (p. 822). A program coordinator expressed a similar challenge in recruiting for their program: “They can’t make it here…it’s transportation or that type of thing” [51] (p. 379).

Availability was the final accessibility barrier described in 11 barriers across 10 studies [24, 37, 39, 43, 45, 63, 65, 70, 71, 73]. Incompatibility of patient schedules with exercise program offerings was the most common concern, especially when programs offered fixed schedules [39, 42, 63, 65, 73], and this was important for patients receiving treatment [39, 71]. For instance, one patient aged 51 commented: “There were two exercise sessions per week…one of them was my treatment day so I had to rule it out altogether” [39] (p. 1291). The inability to attend because programs were “fully booked” [39] (p. 1291) was also noted.

### Patient

Twenty-five barriers were described in 15 studies [37, 41, 42, 45, 50, 53, 67, 70, 72, 74–79]. Patient barriers were described in relation to their knowledge about exercise.

#### Knowledge

All studies at the patient level illustrated a lack of understanding about exercise [37, 41, 42, 45, 50, 53, 67, 70, 72, 74–79]. Patients described not knowing they should [70, 76] or could [50] exercise, not knowing how to exercise [41] or not being made aware of available programs [39, 45, 76].

Patients reported wanting specific advice from a medical professional [53, 77], yet in eight studies [37, 39, 42, 53, 74, 75, 78, 79], concerns were raised about the utility of the advice received from HCPs 13 times, describing it as “not specific” or “vague”: “…they say to keep active in doing what you’re doing, and so that’s what I do” (65 + patient) and “[the oncologist] didn’t really talk to me [about exercise]. He said it’s best and I took it upon myself” (younger than the 65-year-old patient) [75] (p. 90). One study [41] reported that 20 percent of the 834 included patients (n = 167) that indicated contradictory information about exercise made them unsure how to be physically active and another study [53] reported patients being instructed to reduce or “not worry” about exercise when asking their doctor.

### Economic and political context

Seventeen barriers were described in 11 studies [34, 35, 40, 42, 43, 48, 49, 57, 58, 62, 63]. Economic and political context barriers were described regarding policies and financial arrangements.

#### Policies and financial arrangements

A lack of standard policies directing the inclusion of exercise into care was reported as a barrier (n = 11) in seven studies [34, 48, 49, 56–58, 63] and the lack of structured reimbursement policies for exercise (n = 6) across four [40, 42, 43, 62]. As described by Rogers and colleagues, these gaps impacted the care offered to patients because the majority of inactive patients are not “complex” enough to meet the medical requirements for a referral to physiotherapists or occupational therapists [57] (p.822).

### Social context

Fifteen barriers were described in 10 studies [24, 34, 38, 43, 48, 51, 52, 55, 57, 58]. Social context barriers were described with regard to collaboration and leadership.

#### Collaboration and leadership

Thirteen collaboration barriers were identified in eight studies [34, 38, 42, 43, 48, 51, 57, 58]. Poor interprofessional communication and collaboration, specifically between the oncology teams and other HCPs (including GPs and allied health professionals), was a concern [42, 43, 48, 51, 58]. Poor communication was also noted between HCPs and exercise program coordinators [51]. Nevertheless, there was a recognition that more collaboration was required to ensure that exercise was incorporated into care [57]. The quote below illustrates the challenge of collaboration expressed across the studies.

*I do think it probably is part of our role to be doing that but I don’t think it’s solely our role…we don’t always get to clinics to see patients for a follow-up, so consultants have to…take some of that responsibility as well…* (colorectal cancer nurse specialist) [57] (p. 819).

Two studies [24, 52] specified lack of leadership support as an issue impeding the integration of exercise into oncology care, noting pushback because exercise initiatives were perceived as “unsafe” and “expensive to coordinate” [24] (p. 3120).

*You have to have support from the upper end, the decision makers in order for any of this to even happen, you know minus all the barriers with health professionals and the actual participants themselves and what not. If you do not have funding and the support, then it’s not going to happen *[52].

## Discussion

This systematic scoping review synthesized 243 reported barriers impeding implementation of exercise into routine care for cancer patients derived from 50 original research studies. Using an ecological framework [26], a comprehensive understanding of the challenges across all six levels of healthcare is presented. Three key issues were revealed as a result of this work. First, the therapeutic potential of exercise in cancer care is generally recognised by patients and HCPs; however, barriers exist at every level of healthcare to impede its implementation into routine cancer care. These barriers are interrelated, and, consequently, solving one on its own will not be enough to create meaningful progress. Next, the largest concentration of barriers exists at the organizational level of healthcare. Structures and resources are not in place to support an exercise prescription or referral. These organizational challenges are central to all implementation solutions. Finally, implementation in exercise oncology is complex. Solutions will require input from multiple stakeholders across every level of a healthcare system. Sharing experiences of how implementation is being approached in a variety of settings will be invaluable as this nascent field continues to evolve.

In the articles reviewed here, implementation barriers for exercise in oncology care were well described from HCP and patient perspectives suggesting exercise is a recognised therapy among these stakeholders. Our search yielded 50 studies representing 1895 people with cancer and 2846 HCPs from around the world. The abundance of studies exploring patient and provider barriers demonstrates that the role for exercise in oncology is well recognised, but its implementation in routine cancer care remains a challenge. Moreover, implementation is clearly a topic of interest in the field given that most (70%) studies were published since 2017. Our findings support the literature describing HCPs acceptance of the therapeutic benefits of exercise [2, 18, 47], but note challenges to its implementation across all six levels of healthcare.

The organizational level of healthcare accounted for the highest number of reported barriers 38% (n = 93), nearly double that of any other level (Fig. 2). Given the general acceptance of exercise as a therapy in cancer care [2, 18, 47], this finding suggests that a specific focus on overcoming organizational level barriers is needed to close the research to practice gap in exercise oncology. Inadequate structures to support the inclusion of exercise into care was the most frequently reported barrier in the review (n = 38), followed closely by a lack of staff and/or resources to build exercise into care (n = 34). The concentration of barriers in these areas demonstrates a need to support all efforts to integrate exercise into care with concurrent operational changes. For example, efforts to help HCPs overcome their lack of knowledge (n = 23) or change their attitude toward discussing exercise (n = 24) will not be useful if they are not accompanied by a solution to either increase the time allotted for HCPs to spend with patients or to create an established referral pathway to a qualified exercise professional.

Organizational stakeholders are critical to the operational change efforts required for implementation of exercise oncology into practice, yet their perspective was largely absent in our findings. Patients and HCPs accounted for 99% of all participants across the 50 reviewed studies, whereas organizational stakeholders accounted for fewer than 1%. This absence is a concern because organizational leaders are often the gatekeepers for system changes and are responsible for the cultural shifts within an organization that are necessary to adopt new practices [80]. Understanding their perspective is critical to develop meaningful change strategies as it often differs from stakeholders at other levels of healthcare [81]. Moreover, organizational stakeholders’ input regarding potential strategies for change offers a real-world perspective that accounts for the practical needs of running a business. Their engagement is critical in working toward actionable solutions to integrate exercise into cancer care. Implementation research in exercise oncology should adapt to include organizational stakeholders. First, researchers should conduct exploratory work to articulate the barriers to action of this poorly understood sector. Second, researchers should include organizational stakeholders in the planning stages of projects to ensure that the research questions and design will lead to outcomes that are relevant and actionable for organizations.

For exercise to be a meaningful part of routine care, programs need to be accessible to patients, yet the second most frequently reported barrier in our review described challenges related to cost, location and availability of exercise (n = 36). These concerns created hesitation among HCPs to offer exercise and among patients to participate in available programs [39, 53, 57, 71, 73]. Economic and political barriers (n = 17) augment these noted accessibility challenges. For example, patients expressed concerns about the direct costs of exercise participation (n = 11). Cost issues are underpinned by a noted lack of standard reimbursement policies for exercise (n = 6). Most exercise professionals are not covered by traditional healthcare benefits [82, 83], and when they are covered, the process to obtain reimbursement is often complex and the coverage not sufficient [84]. This lack of meaningful financial compensation for exercise not only limits patient participation, but it also limits an organization’s ability to offer exercise programming and serves as a deterrent to potential exercise professionals who struggle to find sustainable employment opportunities as their services are not considered billable. Working toward policy changes that incorporate exercise as part of traditional medical systems has the potential to concurrently address barriers across multiple levels of healthcare.

Despite their reported lack of knowledge (n = 24) regarding exercise prescription and the known lack of referral pathways, implementation initiatives continue to call on HCPs to address exercise with patients because of the known role oncology clinicians have in shaping a patient’s health behaviours [85]. While HCPs are doing their best to fulfil this role, the resultant vague exercise advice does not appear to be helping patients change their behaviour (n = 13). As the field works to make meaningful changes to practice, it is important that HCPs do not become complacent thinking that this general approach is sufficient. The clinical environment needs to be enhanced, so all care givers can work to their best skillset, creating clear pathways that allow oncology clinicians to connect patients with an exercise professional. Research needs to track the impact of HCPs advice, and any attempts to create a referral pathway to ensure patients are being effectively connected with exercise. It is especially important to ensure calls for change can produce the intended results, given how hard it is to change practices in healthcare [86].

While relatively few barriers (n = 10) were identified that questioned the advantage of utilising exercise in practice, the concerns that were raised highlight a fundamental issue regarding the research to practice gap in exercise oncology: Despite their recognition of the potential benefits of exercise, HCPs remain skeptical of the need to integrate exercise into patient care [38, 43, 62]. A disconnect between how oncology clinicians and researchers perceive the role for exercise in care was illustrated by Fitzpatrick and colleagues [62] in their survey showing, on average, that oncologists’ (n = 38) level of agreement was much lower than that of researchers (n = 20) with the concept that exercise should be part of standard care. Recognition of this mismatch of opinions, combined with the barriers noted regarding HCPs’ lack of awareness about the exercise guidelines [18, 57], suggests that researchers and HCPs should aim for more interdisciplinary approaches in both research and practice. It is critical to ensure that everyone is on the same page about the role of exercise during treatment, as it differs from the role of exercise during other phases of the cancer continuum. Exercise during active therapy should be targeted to meet a patient’s specific challenges. A qualified exercise professional with expertise in oncology is generally required to provide these detailed prescriptions. A level of trust and recognition between researchers, exercise professionals, and clinicians needs to be established to move the field forward, as clinicians have a duty of care to their patients. Moreover, the perception that exercise research is inadequate [39] reinforces the need to explore implementation issues hindering the potential of exercise oncology programs. Effective programs can underperform if they are not implemented well [87].

Finally, implementation in exercise oncology is a complex issue and requires a different approach to traditional clinical research methodologies. As we are in the very early stages of understanding the field of implementation in exercise oncology, it is necessary to share experiences of how programs were designed, created and implemented to help create a road map for others and begin the process of identifying best practices. For example, work by Santa Mina and colleagues describing the development and implementation of an integrated cancer program and offering insights based on their experience [88, 89], and the subsequent work detailing their practical approach to include exercise in the electronic medical records systems [90], offers valuable strategies for others working toward the same goal. As the field is still in its infancy, implementation work in exercise oncology should aim to be transparent by sharing experiences during all phases of the implementation process (i.e. pre-implementation, active implementation and maintenance). Moreover, solutions should be co-created by representatives from multiple stakeholder groups.

### Strengths and limitations

To our knowledge, this is the first study to comprehensively describe implementation barriers in exercise oncology across stakeholder groups and levels of healthcare. While the studies included in this review were not designed to evaluate implementation barriers directly, the scoping methodology ensured a broad and robust range of results. Moreover, the qualitative nature of many studies provided useful insight to the findings. There is risk of bias given the subjective nature of the analysis; however, steps were taken to minimize this risk including use of the ecological framework and multiple authors to review the findings. While the search strategy was not limited by region, the included studies were largely confined to North America and Australia. As a result, the implications may not directly translate to other countries. We recognize limiting the studies to those published in 2010 or later risks not capturing the earliest examples of implementation; however, preliminary searches that included older studies yielded few results and none that were directly relevant to the research question. Additionally, no formal quality scoring for retained articles was conducted as this is not a component of a scoping review. Given the considerable agreement in findings across the large volume of included studies, despite the range of methodologies used, we feel comfortable that any methodological concerns will have minimal impact on the overall results. Finally, this review focuses on barriers to implementation. Future work should explicitly explore implementation facilitators as they are not simply the inverse of barriers and will offer important insight to move exercise oncology research into practice.

## Conclusion

Implementing exercise into routine cancer care is hindered by a web of interrelated challenges across all levels of the healthcare system. These challenges limit the ability of patients to access effective exercise resources during cancer treatment. Organizational barriers are central to most issues, yet the perspectives of organizational stakeholders are largely absent from the literature. Future work should use a multi-level, interdisciplinary approach to explore best practices for overcoming implementation barriers, with organizations as a central focus.

## Supplementary Information

Below is the link to the electronic supplementary material.

Supplementary file1 (DOCX 14 KB)

Supplementary file2 (DOCX 29 KB)

Supplementary file3 (DOCX 20 KB)

## Data Availability

Data sharing is not applicable to this article as no new data were created or analysed in this study.
