# Supplementary material for: Implementation barriers to integrating exercise as medicine in oncology: an ecological scoping review
Source: J Cancer Surviv. 2021 Sep 12;16(4):865–81. doi: 10.1007/s11764-021-01080-0 (PMC9300485; doi:10.1007/s11764-021-01080-0)
Supplement: Supplementary file 1 — Supplementary file1 (DOCX 14 KB) [file 11764_2021_1080_MOESM1_ESM.docx]

Supplemental Table 1
Search terms

| 1 | oncolog* or cancer (title) |
| --- | --- |
| 2 | exercise or physical activity (title) |
| 3 | knowledge translation or implement* or translat* or integrat* or barrier* or facilitator* or enabler* |
| 4 | healthcare or health care or professional practise or professional practice or oncologic care or oncology care or health services or health facilities or medical or clinical care |
| 5 | 1 and 2 and 3 and 4 |

* Truncation
