# Supplementary material for: Implementation barriers to integrating exercise as medicine in oncology: an ecological scoping review
Source: J Cancer Surviv. 2021 Sep 12;16(4):865–81. doi: 10.1007/s11764-021-01080-0 (PMC9300485; doi:10.1007/s11764-021-01080-0)
Supplement: Supplementary file 2 — Supplementary file2 (DOCX 29 KB) [file 11764_2021_1080_MOESM2_ESM.docx]

Supplemental Table 2
Participant information

| Author & Year | Participants (n) | Demographics (Sex, Mean Age (SD)) | Cancer Type | Treatment Status (patients only) |
| --- | --- | --- | --- | --- |
| Agasi-Idenburg et al. 2020 (70) | Patients (n=15) | Females (36%, n=4); Age 72.7 (4.39) | Colorectal (100%, n=15) | Not reported |
|  | Physiotherapists (n=9) | Females (56%, n=5); Age 42 (9.96) |  | N/A |
| Beidas et al. 2014 (35) | Physicians (n=7)  Physiotherapists (n=10)  Nurse practitioners (n=2) | Females (84%, n=16); Age * | Breast cancer | N/A |
| Blaney et al. 2010 (71) | Patients, survivors (n=12)  Patients, palliative (n=10)  Patients, recently diagnosed (n=4) | Females *; Age * | Breast (46.3%, n=12)  Prostate (19.3%, n=5)  Colorectal (3.8%, n=1)  Cervical (3.8%, n=1)  NHL (3.8%, n=1)  Lymphoma (3.8%, n=1)  Bladder (3.8%, n=1)  Leukemia (3.8%, n=1)  Missing (3.8%, n=1) | On active treatment (26.9%, n=7) |
| Bourke et al. 2018 (55) | Patients (n=26) | Female (0%, n=0);  Age >60 (92.3%, n=24) | Prostate (100%, n=26) | On active treatment (100%, n=26) |
|  | Consultant urologists (n=9)  Oncologists (n=10)  Clinical nurse specialists (n=6)  General practitioners (n=3)  Physiotherapists (n=3)  Exercise specialists (n=2)  Service managers (n=1)  Clinical commissioners (n=3)  Primary care physicians (n=1) | Female *; Age * |  | N/A |
| Brunet et al. 2013 (74) | Patients (n=9) | Female (100%, n=9);  Age* | Breast (100%, n=9) | On active treatment (0%, n=0) |
| Bultijnck et al. 2018 (60) | Physiotherapists (n=98) | Female *; Age * | Prostate | N/A |
| Cantwell et al. 2018 (36) | Clinical nurse specialists (n=18)  Radiation oncologists (n=12)  Medical oncologists (n=12)  General practitioners (n=7)  Oncology liaison nurses (n=8)  Breast surgeons (n=5)  Colorectal surgeons (n=5)  Research nurses (n=7)  Advanced nurse practitioners (n=4)  Physiotherapists (n=2)  Plastic surgeons (n=1)  Clinical nurse managers (n=1)  Nurses (n=6)  Radiation therapists (n=1)  Other (n=2) | Female (100%, n=67.0%);  Age >50 (18.7%, n=17) | Multiple (no breakdown given) | N/A |
| Cantwell et al. 2020 (67) | Patients (n=41) | Female (56%, n=23);  Age >50 (82.9%, n=34) | Breast (41%, n=17)  Prostate (27%, n=11)  Colorectal (20%, n=8)  Lung (5%, n=2)  Cervical (2%, n=1)  Melanoma (2%, n=1)  Leukemia (2%, n=1) | On active treatment (4.9%, n=2) |
| Cheville et al. 2012 (75) | Patients (n=20) | Female (50%, n=10); Age 65.4 (8.68) | Stage IIIB or IV non-small cell lung cancer (100%, n=20) | On active treatment (60%, n=12) |
| Culos-Reed et al. 2019 (65) | Patients (n=11) | Female (0%, n=0);  Age >60 (100%, n=11) | Prostate (100%, n=11) | Not reported |
| Dalzell et al. 2017 (33) | N/A | N/A | Multiple | N/A |
| Dennett et al. 2017 (63) | Physiotherapists (n=10)  Exercise physiologists (n=4)  Nurses (n=1) | Female *; Age * | Multiple | N/A |
| Dennett et al. 2020 (37) | Patients (n=9) | Female (78%, n=7); Age 67.1 (8.2) | Diffuse large B-cell lymphoma (33%, n=3)  NHL (11%, n=1)  Acute myeloid leukemia (11%, n=1)  Breast (33%, n=3)  Ovarian (11%, n=1) | On active treatment (100%, n=9) |
|  | Nurses (n=13)  Oncologists (n=3)  Haematologists (n=1)  Occupational therapists (n=6)  Physiotherapists (n=1)  Social workers (n=1) | Female (88%, n=22); Age * | Multiple | N/A |
| Fernandez et al. 2015 (76) | Patients (n=29) | Female (93.1%, n=27);  Age >50 (51.7%, n=16) | Breast (58.6%, n=17)  Other (41.4%, n=12) | Not reported |
| Fitzpatrick et al. 2014 (62) | Oncologists, n=38  Researchers, n=20 | Female; Age * | Multiple | N/A |
| Fong et al. 2018 (24) | Medical oncologists (n=10)  Radiation oncologists (n=2)  General practitioners in oncology (n=1)  Primary care physicians (n=2)  Registered nurses (n=8)  Nurse practitioners (n=1)  Surgical oncologists (n=2)  Mammogram techs (n=1) | Female (70.3%, n=19); Age * | Breast | N/A |
| Fong et al. 2018 (32) | N/A | N/A | Breast | N/A |
| Granger et al. 2016 (38) | Respiratory physicians (n=3)  Thoracic surgeons (n=2)  Oncologists (n=2)  Cancer nurses (n=2)  Physiotherapists (n=8) | Female (65%, n=11); Age 34 [28-48]* | Lung | N/A |
| Granger et al. 2019 (64) | Patients (n=7) | Female (71.4%, n=5); Age * | Lung (100%, n=7) | On active treatment (0%, n=0) |
| Hardcastle et al. 2018 (39) | Patients (n=20) | Female (70%, n=14); Age 63.9 (15.29) | Breast (45%, n=9)  Brain (10%, n=2)  Colorectal (10%, n=2)  Melanoma (5%, n=1)  Prostate (5%, n=1)  Tongue (5%, n=1)  Missing (20%, n=4) | Not reported |
| Haussmann et al. 2018 (40) | Outpatient Physicians (n=287)  GP (n=146)  Medical Oncologists (n=28)  Radiation Oncologists (n=23)  Gastroenterologists (n=16)  Urologists (n=34)  Gynaecologists (n=32)  Surgeons (n=4)  Other (n=4) | Female (45.3%, n=130);  Age > 45 (79.4%, n=228) | Breast (76.3%, n=219)**  Prostate (73.5%, n=211)  Colorectal (76.3%, n=219)  Lung (61.7%, n=177)  Other (41.1%, n=118) | N/A |
|  | Nurses, n=388 | Female (81.9%, n=312);  Age > 45 (35.6%, n=138) | Breast (54.4%, n=211)  Prostate (51.5%, n=200)  Colorectal (63.9%, n=248)  Lung (57.2%, n=222)  Other (50.5%, n=196) | N/A |
| Haussmann et al. 2018 (56) | General practitioners (n=10)  Specialised physicians (n=10)  Oncology nurses (n=10) | Female (50%, n=5); Age 48.7 (9.3)  Female (50%, n=5); Age 45.0 (12.9)  Female (90%, n=9); Age 41.6 (12.0) | Breast, prostate, and/or colon (specifics not reported) | N/A |
| Höh et al. 2017 (41) | Patients (n=905) | Female *; Age * | Multiple (specifics not reported) | Not reported |
| Hubbard et al. 2018 (73) | Patients (n=32) | Female *; Age * | Breast (100%, n=32) | On active treatment (78.1%, n=25) |
| IJsbrandy et al. 2019 (42) | Patients (n=34) | Female 64.7%, n=22); Age 60.4 (11.3) | Breast (38.2%, n=13)  Abdominal cavity (32.4%, n=11)  Pelvic cavity (8.8%, n=3)  Haematological (14.7%, n=5)  Bone (2.9%, n=1)  Lung (2.9%, n=1) | On active treatment (32.4%, n=11) |
| IJsbrandy et al. 2020 (43) | Primary Healthcare Professionals  GPs (n=14)  Physiotherapists (n=17) | Female (51.6%, n=16); Age 47.5 (10.8) | Multiple (specifics not reported) | N/A |
|  | Secondary Healthcare Professionals  Physician or Surgeons (n=13)  Paramedics (n=12)  Nurses (n=13)  Other (n=1) | Female (82.1%, n=32); Age 43.2 (10.9) |  | N/A |
| Kang et al. 2014 (72) | Patients (n=427) | Female (37%, n=158);  Age >60 (46.6%, n=199) | Colorectal (100%, n=427) | On active treatment (30.9%, n=132) |
| Karvinen et al. 2012 (44) | Oncology Nurses (n=274) | Female (97.7%, n=260); Age 48.3 (8.9) | Multiple (specifics not reported) | N/A |
| Kennedy et al. 2020 (45) | Patients (n=119) | Female *; Age * | Multiple | Not reported |
|  | Radiation Oncologists (n=7)  Nurses (n=8) | Female *; Age *  Female *; Age * |  | N/A |
| Keogh et al. 2014 (77) | Patients, non-ADT (n=8)  Patients, ADT (n=6) | Female (0%, n=0); Age 65.0 (6.5)  Female (0%, n=0); Age 65.8 (11.3) | Prostate (100%, n=14) | On active treatment (42.9%, n=6) |
| Keogh et al. 2017 (46) | Oncology Nurses (n=119) | Female (96%, n=113);  Age >45 (63%, n=75) | General oncology (41%, n=48)  Gynaecologic (breast, ovary) (18%, n=21)  Haematology (8%, n=9)  Urogenital (prostate, bladder) (6%, n=7)  Palliative care settings (6%, n=7)  Lung (5%, n=6)  Gastrointestinal/Colorectal (5%, n=6)  Other (10%, n=12) | N/A |
| Ligibel et al. 2019 (47) | MDs (n=664)  DOs (n=24)  PhDs (n=36)  PA, NP, DNP, CRNP (n=58)  MPH, MPA, MSW, other grad degree (n=13)  Nurses (n=17) | n=1,067; Female *; Age * | General (21%, n=172)  Breast (34.1%, n=280)  Gastrointestinal (17.2%, n=141)  Leukemia/Lymphoma (6.3%, n=52)  Genitourinary/Gynaecologic (9.8%, n=80)  Paediatric (1.5%, n=12)  Others (10.2%, n=84) | N/A |
| Maxwell-Smith et al. 2017 (78) | Cancer survivors (n=24) | Female*; Age 69.4 (4.2) | Colorectal (100%, n=24) | On active treatment (0%, n=0) |
| Mulcahy et al. 2018 (58) | Physiotherapy cancer service Managers (n=24) | Female *; Age * | Multiple, not specified | N/A |
| Nadler et al. 2017 (18) | Allied health (n=9)  Nurse (n=21)  Physician (n=46)  Radiation therapist (n=44) | Female (72.5%, n=87); Age 48 (24-66) | Breast (60.8%, n=73)  Lung (47.5%, n=57)  GI—lower (45.8%, n=55)  GI—upper (41.7%, n=50)  Head and neck (41.7%, n=50)  Gynaecological (40.8%, n=49)  Central nervous system (37.5%, n=45)  Melanoma and skin (32.5%, n=39)  Sarcoma (31.7%, n=38)  Haematological (30.0%, n=36)  Genitourinary (16.7%, n=20)  General practice (13.3%, n=16) | N/A |
| O’Hanlon et al. 2014 (48) | Physiotherapists (n=26)  Oncology Nurses (n=58) | Female (96.1%, 25); Age *  Female (98.3%, 57); Age * | Multiple (specifics not reported) | N/A |
| Park et al. 2015 (49) | Surgeons (n=41)  Medical oncologists (n=78)  Radiation oncologists (n=25)  Other (n=21) | Female (33.5%, n=56); Age 43.0 (8.6) | Colorectal (22.9%, n=63)  Gastric (18.5%, n=51)  Breast (17.0%, n=47)  Lung (16.7%, n=46)  Liver (9.8%, n=27)  Other (14.9%, n=41) | N/A |
| Patel et al. 2018 (69) | Medical oncologists (n=3)  Urologists (n=4)  Radiation oncologists (n=1)  Oncology nurse specialists (n=1)  Urology nurse specialists (n=1)  Radiation therapists (n=1)  General practitioners (n=2)  Physiotherapists (n=2)  Acupuncturists (n=2) | Female (50%, n=8); Age 49.3 (12.0) | Prostate | N/A |
| Perry et al. 2020 (61) | Survivors (n=61) | n=61; Female (100%, ne=61);  Age 72.4 (9.8) | Breast | Not reported |
|  | Medical oncologists (n=1)  Radiation oncologists (n=1)  Surgical oncologists (n=1)  Nurse practitioners (n=1)  Registered nurses (n=3)  Social workers (n=3)  Provider with admin role (n=1) | N=11; Female (82%, n=9); Age * |  |  |
| Roberts et al. 2019 (57) | Cancer nurse specialists (n=19) | Female (95%, n=18); Age * | Breast (21%, n=4)  Prostate (32%, n=6)  Colorectal (47%, n=9) | N/A |
| Rogers et al. 2019 (66) | Interventionists  Fitness specialists (n=5)  Administrative staff (n=3)  Gym manager, patient navigator or health educator (n=1) | Female (81.8%, n=9); Age 42.3 (15.3) | Multiple (specifics not reported) | N/A |
|  | Community stakeholders  Hospital administrators (n=5)  Healthcare professionals (n=5)  Community organisation representatives (n=4)  Social workers or Occupational therapists (n=3) | Female (78.9%, n=15); Age 49.2 (9.7) |  | N/A |
| Romero-Elias et al. 2020 (50) | Patients (n=10) | n=10; Female (50%, n=5);  Age 58.8 (12.47) | Colorectal | Not reported |
|  | Psycho-oncologists (n=1)  Oncologists (n=4)  Nurses (n=5) | n=10; Female (60%, n=6);  Age 40.8 (7.7) |  | N/A |
| Santa Mina et al. 2015 (51) | Program coordinators cancer-exercise programs | n=13; Female (84.6%, n=11);  Age 42.6 (11.2) | Multiple (specifics not reported) | Not reported |
| Shea et al. 2019 (52) | Oncology providers (n=14)  Allied healthcare providers (n=6)  PA program leaders (n=5)  Administrators (n=5) | Female *; Age * | Multiple (specifics not reported) | Not reported |
| Smaradottir et al. 2017 (53) | Patients  Metastatic (n=9)  Nonmetastatic (n=11) | Female (33.3%, n=3); Age 62 (7.6)  Female (63.6%, n=3); Age 65 (8.1) | Multiple (excluding breast)  Lung (30%, n=6)  Ovarian (10%, n=2)  Pancreas (10%, n=2)  Prostate (5%, n=1)  Rectal (5%, n=1)  Uterine (5%, n=1)  Bladder (5%, n=1)  Chronic lymphocytic leukemia (5%, n=1)  Lung (15%, n=3)  Gastric (5%, n=1)  Hepatic/testicular (5%, n=1) | On active treatment (45%, n=9) |
|  | Healthcare Providers (n=9) | Female (44%; n=4); Age 45 (13) |  | N/A |
| Smith et al 2017 (79) | Patients (n=19) | Female (57.9%; n=11); Age 59 (13.11) | Breast (36.8%, n=7)  Colorectal (5.3%, n=1)  Prostate (5.3%, n=1)  Lung (5.3%, n=1)  Thyroid (10.5%, n=2)  NHL (15.8%, n=3)  Hodgkin lymphoma (5.3%, n=3)  Testicular (5.3%, n=1)  Bladder (5.3%, n=1)  Melanoma (10.5%, n=2)  Neuroendocrine tumour (5.3%, n=1) | On active treatment (0%, N=0) |
| Smith-Turchyn et al. 2016 (54) | Medical oncologist (n=7)  Primary care nurse (n=5)  Radiation oncologist (n=4)  Surgeon (n=2)  Radiation therapist (n=2)  General practitioner in oncology (n=1)  Nurse practitioner (n=1)  Social worker (n=1)  Dietician (n=1) | Female (66.7%, n=16); Age * | Breast | N/A |
| Spost 2015 (59) | Oncologists (n=3)  General practitioners (n=5)  Family medicine physicians (n=15)  Surgical oncologists (n=4)  Other (n=9) | Female (55.6%, n=20);  Age >45 (25%, n=9) | Breast | N/A |
| Sutton et al. 2017 (68) | Patients (n=16) | Female (0%, n=0); Age* | Prostate | On active treatment (18.8%, n=3) |
|  | Consultant urological surgeon (n=3)  Uro-Oncology clinical nurse specialist (n=3)  Cancer support worker/dietician/physiotherapist (n=4) |  |  | N/A |
| Tomasone et al.  2017 (34) | Providers  Administrations  Patients  Academics  Community partners | Female *; Age * | Multiple (specifics not reported) | N/A |

NHL=Non-Hodgkin lymphoma

*median [IQR]

**multiple responses possible
