# Supplementary material for: Implementation barriers to integrating exercise as medicine in oncology: an ecological scoping review
Source: J Cancer Surviv. 2021 Sep 12;16(4):865–81. doi: 10.1007/s11764-021-01080-0 (PMC9300485; doi:10.1007/s11764-021-01080-0)
Supplement: Supplementary file 3 — Supplementary file3 (DOCX 20 KB) [file 11764_2021_1080_MOESM3_ESM.docx]

Supplemental Table 3
Context of barriers identified for each study

| Author & Study Year | I | IP | P | S | O | E/P | TOTAL |
| --- | --- | --- | --- | --- | --- | --- | --- |
| Agasi-Idenburg et al. 2020 | 1 |  | 4 |  |  |  | 5 |
| Beidas et al. 2014 |  |  |  |  | 1 | 2 | 3 |
| Blaney et al. 2010 | 2 |  |  |  |  |  | 2 |
| Bourke et al. 2018 | 1 | 1 |  |  | 1 |  | 3 |
| Brunet et al. 2013 | 1 |  | 1 |  |  |  | 2 |
| Bultijnck et al. 2018 |  |  |  |  | 1 |  | 1 |
| Cantwell et al. 2018 | 2 |  |  |  | 3 |  | 5 |
| Cantwell et al. 2019 |  |  | 2 |  | 1 |  | 3 |
| Cheville et al. 2012 |  |  | 2 |  |  |  | 2 |
| Culos-Reed et al. 2019 | 1 |  |  |  | 2 |  | 3 |
| Dalzell et al. 2017 |  |  |  |  | 3 |  | 3 |
| Dennett et al. 2017 | 2 | 1 |  |  | 1 | 1 | 5 |
| Dennett et al. 2020 | 2 | 1 | 1 |  | 5 |  | 9 |
| Fernandez et al. 2015 |  |  | 3 |  |  |  | 3 |
| Fitzpatrick et al. 2014 | 2 | 1 |  |  | 5 | 2 | 10 |
| Fong et al. 2018 | 1 | 4 |  | 1 | 3 |  | 9 |
| Fong et al. 2018 |  |  |  |  | 2 |  | 2 |
| Granger et al. 2016 | 4 | 2 |  | 1 | 8 |  | 15 |
| Granger et al. 2019 |  |  |  |  | 1 |  | 1 |
| Hardcastle et al. 2017 | 5 |  | 1 |  | 1 |  | 7 |
| Haussmann et al. 2018 |  |  |  |  | 2 | 2 | 4 |
| Haussmann et al. 2018 | 1 | 6 |  |  | 1 |  | 8 |
| Höh et al. 2017 |  |  | 3 |  | 2 |  | 5 |
| Hubbard et al. 2018 | 3 |  |  |  |  |  | 4 |
| IJsbrandy et al. 2019 | 1 |  | 1 | 1 | 2 | 1 | 6 |
| IJsbrandy et al. 2020 | 3 | 2 |  | 5 | 5 | 1 | 16 |
| Kang et al. 2014 | 1 |  | 1 |  |  |  | 2 |
| Karvinen et al. 2012 |  | 1 |  |  | 1 |  | 2 |
| Kennedy et al. 2020 | 2 |  | 1 |  | 1 |  | 4 |
| Keogh et al. 2014 |  |  | 1 |  |  |  | 1 |
| Keogh et al. 2017 |  | 1 |  |  | 2 |  | 3 |
| Ligibel et al. 2019 |  | 1 |  |  | 2 |  | 3 |
| Maxwell-Smith et al. 2017 |  |  | 1 |  |  |  | 1 |
| Mulcahy et al. 2018 |  |  |  | 1 | 4 | 1 | 6 |
| Nadler et al. 2017 |  | 3 |  |  | 1 |  | 4 |
| O’Hanlon et al. 2014 |  | 2 |  | 1 | 5 | 1 | 9 |
| Park et al. 2015 |  | 1 |  |  | 1 | 1 | 3 |
| Patel et al. 2018 |  | 2 |  |  |  |  | 2 |
| Perry et al. 2020 | 1 |  |  |  | 2 |  | 3 |
| Roberts et al. 2019 | 1 | 4 |  | 1 | 2 | 1 | 9 |
| Rogers et al. 2019 |  |  |  |  | 2 |  | 2 |
| Romero-Elias et al. 2020 |  | 1 | 1 |  | 3 |  | 5 |
| Santa Mina et al. 2015 | 3 | 2 |  | 1 | 1 |  | 7 |
| Shea et al. 2019 | 1 | 3 |  | 1 | 2 |  | 7 |
| Smaradottir et al. 2017 | 2 | 2 | 1 |  | 3 |  | 8 |
| Smith et al 2017 |  |  | 1 |  |  |  | 1 |
| Smith-Turchyn et al. 2016 | 2 | 3 |  |  | 4 |  | 10 |
| Spost 2015 |  |  |  |  | 1 |  | 1 |
| Sutton et al. 2017 |  | 1 |  |  |  |  | 1 |
| Tomasone et al. 2017 | 1 | 2 |  | 2 | 6 | 4 | 15 |
| TOTAL | 46 | 47 | 25 | 15 | 93 | 17 | 243 |

I=innovation; IP=individual professional; P=patient; S=social; O=organisational; E/P=economic and political
